# The Influence of Moderate Electroporation on *E. coli* Membrane Permeability

**DOI:** 10.3390/microorganisms13081925

**Published:** 2025-08-18

**Authors:** Ester Bar-Hanun, Ester Hanya, Abhishiktha Chiliveru, Rivka Cahan

**Affiliations:** Department of Chemical Engineering, Ariel University, Ariel 40700, Israel; estibar92@gmail.com (E.B.-H.); esterh@ariel.ac.il (E.H.); abhishikthachilivery@gmail.com (A.C.)

**Keywords:** pulsed electric fields, membrane permeability, *Escherichia coli*, propidium iodide, fluorescein isothiocyanate dextran

## Abstract

This study examined the membrane permeability of *E. coli*, which were exposed to a moderate pulsed electric field (PEF) (3.3 kV/cm). The membrane permeability of *E. coli* was examined as a function of time after exposure to PEF. When comparing the percentage of propidium iodide (PI) permeability at a given time from PEF exposure, it appeared that as the bacterial density increased, there was a decrease in PI permeability. The permeability to PI in the bacterial suspensions of 0.05, 0.1, and 0.5 OD, 90 min after exposure, was 56.4 ± 4.08%, 43.91 ± 0.75%, and 29.47 ± 3.31%, respectively. Membrane permeability was also examined in different phosphate-buffered saline (PBS) concentrations. At 0.05 OD there was a linear correlation between PBS concentrations (0.56, 0.75, and 1 mM) and PI permeability (28.36 ± 2.22%, 61.08 ± 3.17%, and 98.2 ± 0.9%, respectively). At the higher bacterial densities of 0.1 and 0.5 OD, this phenomenon was not evident. Examination of bacterial membrane permeability using 4, 70, and 250 kDa fluorescein isothiocyanate (FITC)-dextran revealed that PEF led to 4kDa FITC-dextran permeabilization of 27.94 ± 3.76%. The PEF parameters used did not influence the bacterial cell size and viability. This study shed light on bacterial membrane permeability as a function of conductivity and bacterial density under PEF exposure.

## 1. Introduction

Pulsed electric field (PEF) technology utilizes short, high-intensity electric pulses that may modify the membrane properties by inducing structural rearrangements in the lipid bilayer. If the electric field strength exceeds a specific threshold, molecules move through the pores according to the gradient across the membrane. PEF may lead to either reversible pores that seal after a short period, allowing the cell to remain viable, or to irreversible pores that result in cell death [1,2,3]. The application of PEF has received attention for bacterial disinfection in the food industry. It is considered a “clean” technology, and in contrast to conventional pasteurization methods, PEF does not require heat, thus preserving the taste, color, and flavor of food [4,5]. Applications of PEF have also been reported for medical applications, such as electrochemotherapy and tumor treatment [6].

The key parameters influencing PEF efficiency are the electric field parameters, cell size and type, and medium conductivity. The electric field strengths required to eradicate bacteria are typically tens of kV/cm. However, higher electric fields, such as 100 kV/cm, and lower levels, such as 4.5 kV/cm, have also been reported [7,8,9,10]. The electric pulse length affects the stability and size of the formed pore. The pulse pattern can be square or exponential; the duration may range from nanoseconds to milliseconds [7,9], and the number of pulses varies between one and tens of thousands. Microbial cell size and type such as bacteria, mold fungi, and vegetal cells, may also influence PEF efficiency [9,11,12,13,14]. It was found that eukaryotes, which have larger cell sizes compared to prokaryotes, tend to show stronger tolerance to PEF. In studies of prokaryotes, it has been observed that Gram-positive bacteria exhibit higher tolerance to PEF compared to Gram-negative bacteria, due to their thicker cell wall structure [15,16]. The cells’ medium conductivity ranges mostly from µS/cm to mS/cm. The medium conductivity is influenced by both the ion concentration and the concentration of microorganisms. Lan et al. (2022) [17] found a correlation between the biomass concentrations of *Saccharomyces cerevisiae* yeast and *Chlamydomonas reinhardtii* microalgae and their ohmic resistance. A decrease in impedance correlates with an increase in the medium conductivity (ohmic resistance) [17]. The medium conductivity influences the distribution of the electric field and heat generation. Multiple studies have demonstrated that the extracellular medium conductivity has a significant influence on the PEF effect, showing a linear correlation between PEF efficiency and medium conductivity [18,19,20]. Ivorra et al. (2010) [21] hypothesized that when an external voltage is applied, part of it drops across the membrane, while the rest drops across the extracellular and intracellular media. The voltage drop at the membrane not only depends on the membrane conductivity but also on the conductivities of the extracellular and intracellular media [21]. PEF technology became an interesting application for inactivating microorganisms in food products. The science behind the transfer of electrical pulses in food is based on the fact that food contains ions that give it a certain level of electrical conductivity [22].

The PEF electric field (3.3 kV/cm) selected for this study was chosen based on prior studies demonstrating effective sublethal membrane permeability in *E. coli* and other bacteria under moderate field intensities. Kuang et al. (2024) showed that PEF (0.24 kV/cm) induced *E. coli* membrane damage and disrupted energy metabolism [23]. Wang et al. (2020) [24] investigated the inactivation of *Bacillus subtilis* spores in sterile distilled water using moderate electric fields (300 V/cm) under various temperatures (<30, 55, 65, and 75 °C). At temperatures below 30 °C, the reduction in spores was 0.6 log. Inactivation induced by the same treatment time increased to 1.8, 2.0, and 2.5 log reductions when the temperatures increased to 55, 65, and 75 °C, respectively [24]. Loghavi et al. (2009) [25] examined the effects of a moderate electric field (2 V/cm in various Hz) on the cell membrane permeability of *Lactobacillus acidophilus.* It was reported that low frequencies of 45 and 60 Hz at the lag phase affected cell membrane permeability with no significant differences in CFU count [25]. Thongkong et al. (2023) improved the extraction yield of rice proteins when using PEF treatment at 2.3 kV for 25 min; the protein extraction increased by 20.71–22.8% [26].

*E. coli* was selected for this study as a well-characterized representative of Gram-negative bacteria, frequently used for examination of membrane permeability, electroporation, and antibacterial studies. The *E. coli* outer membrane is rich in lipopolysaccharides (LPS), which provide an effective barrier to hydrophobic compounds. The structural complexity of *E. coli* makes it an ideal model for studying the effects of PEF treatment on bacterial membrane integrity. Moreover, *E. coli*’s widespread presence is found in both environmental, food industry, and clinical settings [27].

This study examined the membrane permeability of *E. coli* suspensions after being exposed to moderate PEF (3.3 kV/cm at 100 Hz (f)) in rectangular pulses with a duration of 10 µs (τ). The membrane permeability was assessed using PI in *E. coli* suspensions with different optical densities and different PBS concentrations, as a function of time (0, 30, 60, and 90 min) after exposure to the PEF. To evaluate the size of molecules that can penetrate the membrane after PEF treatment, we used the fluorescent substance FITC-dextran with various molecular weights (70, 250, and 4 kDa). The PEF parameters used in this study did not influence the bacterial cell size and viability. However, the membrane permeability was changed as a function of conductivity and bacterial optical density.

## 2. Materials and Methods

### 2.1. Growth Conditions

*E. coli* (25922) was purchased from ATCC (Manassas, VA, USA). The bacteria (0.15 OD, 660 nm) were grown in Brain Heart Infusion (BHI) medium (30 mL in a 150 mL Erlenmeyer flask) (HiMedia Laboratories, Mumbai, India) until the logarithmic growth phase. The growth was carried out in an incubator at 37 °C with shaking at 200 rpm. Quantification of bacterial growth was performed by measuring the optical density of the suspension using a spectrophotometer at 660 nm (Genesys 10S UV-VIS, Thermo Scientific, Waltham, MA, USA).

### 2.2. Characterization of the PEF Treatment

*E. coli* suspensions (350 µL) were subjected to an electric field intensity of 3.3 kV cm^−1^ at 100 Hz (f), rectangular pulses with a duration of 10 µs (τ). A total of 5000 pulses (n) were delivered, divided into 10 trains of 500 pulses each. Each train lasted for 5 s, with a 2 s interval between trains. The chamber voltage (UCH) polarity was alternated for each train, ensuring that the subsequent train operated with the opposite polarity [28]. Schematic drawings of the high-voltage generator and the electronic circuit, as well as the electroporator chamber, are shown in Supplementary Material (SM) File S1. Photos of the electroporator chamber and the instrument are shown in SM File S2. The PEF treatment was performed at different bacterial densities and various PBS concentrations. A 10 mM PBS stock solution (containing 1.47 mM KH_2_PO_4_, 8.1 mM Na_2_HPO_4_, 2.67 mM KCl, and 136.9 mM NaCl) was purchased from Biological Industries (Kibbutz Beit HaEmek, Israel). Various PBS concentrations (0.56–2 mM) were prepared by diluting the stock solution with ultrapure (UP) water [13].

### 2.3. Examination of the Bacterial Membrane Permeability and Cell Size

*E. coli* bacterial cells were grown in a BH medium until the logarithmic phase, followed by centrifugation and suspension in PBS concentrations as indicated for each experiment. The *E. coli* suspensions were prepared to bacterial optical densities as described in each experiment. The bacterial suspensions (350 µL) were exposed to PEF as described in Section 2.2.

For membrane permeability examinations, the PEF bacterial suspension (150 µL) was then stained with propidium iodide (PI) (Sigma-Aldrich, St. Louis, MO, USA) to a final concentration of 1.5 µM. Immediately after adding the PI, the samples were incubated at 37 °C for 5 min. The control *E. coli* suspensions were prepared in the same manner as the PEF-treated cells, except for the exposure. The PI membrane permeability was examined using a CytoFLEX flow cytometer (FACS) (Beckman Coulter, Atlanta, GA, USA). The cell size of the PEF-treated bacteria and the control bacteria were examined using forward scatter (FSC) analysis. FSC is related to the cell’s diameter and illustrates relative cell size. There is a linear correlation between FSC values and cell size. Data were analyzed using FlowJo_10.10 software (Tree Star, San Carlos, CA, USA). Each sample included about 50,000 bacterial cells [13].

### 2.4. Examination of the Membrane Permeability to Different Molecule Sizes

A solution of fluorescent FITC-dextran dye (Sigma-Aldrich, St. Louis, MO, USA) (10 µL) with different molecular weights of 4, 70, and 250 kDa (at a final concentration of 1 mg/mL) was added to a bacterial suspension (150 µL) that had been exposed to PEF as described in Section 2.2. Immediately after exposure to FITC-dextran, the samples were incubated at 37 °C for 0, 30, 60, and 90 min, followed by centrifugation (21,200× *g* for 10 min). The resulting bacterial pellet was resuspended in 1 mM PBS (this process was performed twice), and the samples were loaded into a cell and tissue culture plate (96-well) (JET BIOFIL, Alicante, Spain). Each sample was analyzed to confirm approximately 50,000 bacterial cells. The same process was performed for the bacterial suspension that was not exposed to PEF (control sample) [29].

### 2.5. Viable Count Assay

A portion (100 µL) of the PEF-treated suspension was serially diluted, and the appropriate dilutions were spread onto BH agar plates followed by incubation at 37 °C for 24 h. The number of viable cells was determined by counting the colony-forming units (CFU), adjusted for the respective dilution factors. The CFU count was calculated per 1 mL. The same procedure was performed for the untreated PEF samples (control).

### 2.6. The Conductivity of the Buffer in the Bacterial Suspension

The conductivity of *E. coil* suspensions in PBS (0.125, 0.25, 0.5, 1, and 2 mM) corresponding to optical densities of 0.05, 0.1, and 0.5 OD (600 nm), and PBS without bacteria in each of the indicated concentrations, were measured using a Multi-Parameter Analyzer, DZB-712 (Nanbei Instruments, Yantai, China). The conductivity values are shown in Table 1.

### 2.7. Statistical Analysis

The data were analyzed using Prism version 10 (GraphPad, Boston, MA, USA). Data normality was evaluated with the Shapiro–Wilk test, while the Brown–Forsythe test was used to verify the homogeneity of variances. A two-tailed one-way ANOVA was performed to assess differences among multiple groups, followed by the Bonferroni post hoc test. Results are presented as the mean and standard deviation (SD) of n ≥ 3. All statistical analyses were conducted at a significant level of α = 0.05.

## 3. Results and Discussion

### 3.1. Membrane Permeability at a Bacterial Density of 0.05–0.5 OD, as a Function of the Time After PEF Exposure

**Figure 1:**
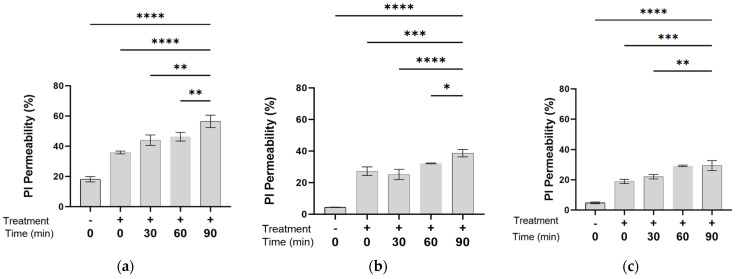
PI permeability as a function of time after exposure (0, 30, 60, and 90 min) to PEF in *E. coli* suspensions with optical density of 0.05 OD (**a**); 0.1 OD (**b**); and 0.5 OD 600 nm (**c**). An ANOVA test was conducted to ascertain statistical significance. The data represent the mean ± SD of n = 3, * *p* < 0.05, ** *p* < 0.01, *** *p* < 0.001, and **** *p* < 0.0001 for multiple-variable comparisons.

*E. coli* bacterial cells were grown in a BH medium and prepared for PEF treatment as described in Section 2.3, followed by centrifugation, and suspension of the sediment in PBS (0.56 mM) to optical densities of 0.05, 0.1, and 0.5 OD (600 nm). The bacterial suspensions were exposed to PEF as described in Section 2.2. The PI permeability was tested at different time points after exposure (0, 30, 60, and 90 min) using FACS analysis (Figure 1a–c). The permeability percentage of the PEF-treated cells was compared to that of control bacterial suspensions, which were treated the same but not exposed to PEF.

Figure 1a shows that in a bacterial suspension of 0.05 OD, there was a gradual increase in PI permeability over time after PEF exposure. The highest percentage of PI permeability, 56.4 ± 4.08%, was found 90 min after the exposure to PEF. The PI penetration percentage obtained immediately after the exposure (time +0) was 35.9 ± 0.96%, whereas PI penetration in a bacterial suspension that was not exposed to PEF (−0) was only 18.14 ± 1.73%. In a bacterial suspension of 0.1 OD (Figure 1b), the highest PI permeability also occurred 90 min after exposure to PEF, but the value was 43.91 ± 0.75%, lower than the permeability in a bacterial suspension of 0.05 OD. Regarding the function of time after exposure, the increased PI permeability in the bacterial suspension with 0.1 OD was not as significant as that seen in the 0.05 OD specimen. In the case of 0.5 OD (Figure 1c), the highest bacterial density which was examined in this study, as well as the highest bacterial permeability (29.47 ± 3.31%), was also achieved 90 min after PEF exposure. In this case, unlike the lower bacterial densities of 0.1 and 0.5 OD (600 nm), there was no significant change in penetration percentage between 60 and 90 min.

When comparing the percentage of permeability at the same time lapses after PEF exposure, it appeared that as the bacterial density increased, there was a decrease in PI permeability. For example, the permeability 90 min after exposure was 56.4 ± 4.08% at a bacterial density of 0.05 OD, 43.91 ± 0.75% at 0.1 OD, and only 29.47 ± 3.31% at 0.5 OD. In all the bacterial suspensions, the samples that were not exposed to PEF showed the least PI permeability.

### 3.2. The Effect of PEF on Membrane Permeability as a Function of Different PBS Concentrations

**Figure 2:**
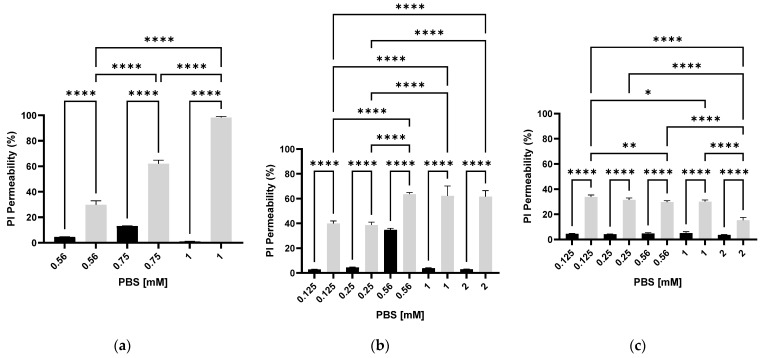
PI permeability as a function of *E. coli* suspensions (0.05 OD (**a**); 0.1 OD (**b**); and 0.5 OD (**c**) 600 nm) in various PBS concentrations (0.125 mM and 2 mM). Bacterial suspensions that were exposed to PEF (gray column) and control bacterial suspensions that were not exposed to PEF (black column). An ANOVA test was conducted to ascertain statistical significance. The data represent the mean ± SD of n = 4. * *p* < 0.05, ** *p* < 0.01 and **** *p* < 0.0001 for multiple-variable comparisons.

To improve PI membrane permeability, *E. coli* bacteria with varying densities (0.1, 0.5, and 0.05 OD 600 nm) were suspended in increasing PBS concentrations (0.125, 0.25, 0.56, 1, and 2 mM). A 350 µL portion of each bacterial suspension was inserted into the electroporator cell and exposed to PEF. After 90 min, PI was added for 5 min. Figure 2a shows the results of PEF treatment in bacterial suspensions with an optical density of 0.05 OD, which were suspended in 0.56, 0.75, and 1 mM PBS. Figure 2b,c show the results of bacterial suspensions with optical densities of 0.1 OD and 0.5 OD, respectively, when suspended in 0.125, 0.25, 0.56, 1, and 2 mM PBS. Control bacterial samples were treated the same as the PEF samples but without exposure to PEF.

Figure 2a shows that the highest PI permeability (98.2 ± 0.9%) was obtained at a bacterial density of 0.05 OD (600 nm) when the PBS concentration was 1 mM. Lower PBS concentrations led to a decrease in permeability. A linear correlation was observed between PBS concentration and PI permeability. In 0.56 and 0.75 mM, the permeability was 28.36 ± 2.22% and 61.08 ± 3.17%, respectively. Figure 2b shows the PI permeability when the bacterial optical density was 0.1 OD (600 nm). The highest PI permeability was obtained when the bacteria were suspended in higher PBS concentrations of 0.56, 1, and 2 mM, with an average permeability of 61.7 ± 4.7%. In the lower PBS concentrations (0.12 and 0.25 mM), the average PI permeability was 36%. Figure 2c shows that the highest PI permeability (33.81 ± 1.85%) at a bacterial density of 0.5 OD 600 nm was when the PBS concentration was 0.125–1 mM. At a PBS concentration of 2 mM, permeability was reduced to 15.37 ± 2.13%.

In summary, the results showed that at a relatively low optical density (0.05 OD) a linear correlation existed between PBS concentrations and PI permeability. At the higher bacterial densities of 0.1. and 0.5 OD, this phenomenon was not so clear; we assume that at these concentrations, the bacteria themselves disrupted the PEF exposure. At all PBS concentrations, a significant difference was observed in the PI membrane permeability of cells exposed to PEF compared to control cells that were not exposed. The variation in the PI permeability of the PEF-nontreated (control samples) may depend on cell physiology, as the experiments were performed on different days with different bacterial cultures.

To evaluate moderate PEF on bacterial cell membrane integrity, fluorescent molecules are used. PI, a small (668 Da) hydrophilic fluorescent molecule, is known to enter the cells when cell integrity is disrupted. Since PEF treatment leads to pore formation, PI can enter the cells and bind to nucleic acids, resulting in a 20- to 30-fold increase in red fluorescence inside the cell compared to nontreated cells. Many studies have demonstrated the reversible and irreversible formation of pores in both Gram-negative and Gram-positive bacteria using PI under various physical and environmental conditions [30,31]. The permeability of molecules out of or into cells was assessed to investigate the effect of PEF on pore formation under. Emanuel et al. (2019) [28] demonstrated that the efficiency of bacterial eradication is not only a function of the electric field strength but is also significantly influenced by the current density. They showed that for *Pseudomonas putida* F1, an increase in current density led to higher membrane permeability. The percentage of PI permeability in an electric field of 1 kV/cm at a current density of 5.2 ± 0.5 A/cm^2^ was 65 ± 0.3%, compared to only 10 ± 0.9% in the control group. Similar permeability (62 ± 2.2%) was observed in an electric field of 2 kV/cm with a lower current density of 3.5 ± 0.3 A/cm^−2^ [28]. This highlights the crucial role of current density in determining the extent of membrane disruption during PEF treatment.

Other molecules besides PI were investigated to examine membrane permeability as a function of PEF treatment. Stirke et al. (2019) [32] examined the accumulation of lipophilic tetraphenylphosphonium ions (TPP+) in yeast cells. The TPP+ absorption was found to be time-dependent, in three stages. Stage I, lasting ~10 s, appeared immediately after the addition of the yeast cells into the TPP+ solution. Stage II, lasting about 3–5 min, commenced when the concentration of TPP+ was about 10%, and Stage III occurred when slow TPP+ absorption took place and saturation was reached, after ~2 h [32].

Gančytė et al. (2023) [33] investigated the effect of media osmolarity on the viability of PEF-treated yeasts. The cells were transferred 5 s after PEF treatment, into either hyperosmotic conditions (1.375 M or 1.75 M sorbitol) or hypoosmotic conditions (0.25 M or 0.625 M sorbitol) and then incubated for 5 min. The results demonstrated that increasing the osmolarity led to a higher count of viable yeast cells. Hyperosmotic conditions retained the yeast cell viability, compared to cells incubated under iso-osmotic conditions. They concluded that hyperosmotic conditions reduced the impact of PEF treatment on yeast viability [33].

The study by Orlacchio et al. (2023) [34] demonstrated that the conductivity and composition of the buffer significantly impact the efficiency of PEF in treating cancer cells. Their results showed that a low-conductivity sucrose-containing buffer (0.2 S/m) minimized the temperature increase during PEF exposure, thereby maintaining cell viability and growth. In contrast, a high-conductivity buffer (1.4 S/m) led to significant temperature elevation, which enhanced cellular membrane permeabilization and reduced cell viability. Specifically, exposure to 500 pulses at an intensity of 50 kV/cm resulted in a significant reduction in spheroid viability. Additionally, local thermal effects contributed to cellular damage, as temperatures exceeding 50 °C caused noticeable deterioration in cell morphology and growth. These findings highlight the importance of precise control over electric field parameters and thermal conditions to achieve selective effects on living cells, underscoring the potential of nanosecond PEF as a noninvasive modality for targeted cancer therapy. These findings also align with previous studies, which indicated that increased buffer conductivity can lead to higher heat generation during PEF treatment, thereby enhancing the permeabilization effect [34].

Nesin et al. (2011) [35] investigated the effects of 60 and 600 ns electric pulses on nanopore formation. The size of opened pores was estimated by iso-osmotic replacement of bath salts (NaCl) with polyethylene glycols and sugars. These solute exchanges showed that for the same integral area of pores opened by 60 and 600 ns PEF treatments, the pore sizes were similar. However, the 600 ns exposure led to higher cell uptake of PI. It was assumed that 600 ns electric pulses opened a greater number of pores smaller than 0.9 nm in diameter [35].

### 3.3. Examination of Bacterial Membrane Permeability with Different Molecular Weights of FITC-Dextran

Fluorescein isothiocyanate (FITC) is composed of FITC-dextran molecules with different sizes, which have been used to estimate microalgae pore size [29]. FITC has excitation and emission spectrum peaks of 495 nm and 519 nm, respectively, giving it a green color. The different sizes of the FITC-dextran molecules are obtained by varying the hydrophilic polysaccharide dextran molecular weight, which is composed of many glucose molecules linked by α-1,6 glycosidic linkages. For example, Neubacher et al. (2014) examined the permeability of FITC-dextran (70 kDa) through pores induced by melittin, an antimicrobial peptide [36,37,38].

**Figure 3:**
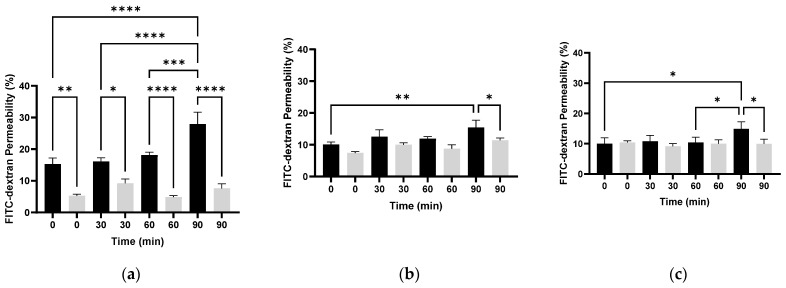
The percentage of FITC-dextran permeability with different molecular weights as a function of the time after exposure to PEF. FITC-dextran of 4 kDa (**a**), 70 kDa (**b**), and 250 kDa (**c**); PEF-exposed *E. coli* (0.05 OD 600 nm) (black column), control sample (not exposed to PEF) (gray column). An ANOVA test was conducted to ascertain statistical significance. The data represent the mean ± SD of n = 4. * *p* < 0.05, ** *p* < 0.01, *** *p* < 0.001, and **** *p* < 0.0001 for multiple-variable comparisons.

To evaluate the molecular size that can penetrate the membrane after PEF treatment, we used the fluorescent substance FITC-dextran with varying molecular weights (250, 70, and 4 kDa). *E. coli* bacteria in the logarithmic phase were suspended in 1 mM PBS to an optical density of 0.05 OD (600 nm). The bacterial suspension was introduced into the electroporator cell and exposed to PEF. After exposure, the cells were incubated with FITC-dextran for 0, 30, 60, and 90 min. Immediately after that, the cells were examined by FACS as described in Section 2.4. The percentages of FITC-dextran permeability with different molecular weights are shown in Figure 3a–c.

The results in Figure 3a showed that the highest percentage of 4 kDa FITC-dextran permeability was obtained in PEF-treated cells when the post-exposure time was 90 min (27.94 ± 3.76%). At all times, there was a significant difference in 4 kDa FITC-dextran permeability in PEF-treated cells, compared to those not exposed to PEF.

In Figure 3b, the highest permeability of 70 kDa FITC-dextran was also observed when this molecule was added to the cells after 90 min, similarly to the 4 kDa FITC-dextran. However, the percentage of penetration was about 2-fold less (15.42 ± 2.3%) than for the 4 kDa molecule. Each time 70 kDa FITC-dextran was added, there was a significant difference between the PEF-treated cells and those that were not treated. However, the increase in permeability over time was not as significant as for the 4 kDa molecule.

The results in Figure 3c showed that the permeability of 250 kDa FITC-dextran remained almost unchanged over time until 60 min. After 90 min, a significant permeability was observed compared to the nontreated and all other treated cells.

In summary, we found an inverse correlation between the molecular weight of FITC-dextran and the percentage of membrane permeability. In *E. coli* bacteria, the penetration percentage was higher as the molecular weight of FITC-dextran was lower.

Smith et al. (2014) [39] illustrated pore formation in a cell system model as a response to an electric field of 1 kV/cm via a 100 μs trapezoidal pulse. Early in the pulse, rapid pore creation occurred, followed by gradual formation of a subpopulation of large pores (~30 nm radius). After the pulse, small pores (~1 nm radius) appeared, which slowly decayed. This phenomenon was also observed with pulses 100 ns to 1 ms in duration. With each applied field strength, a total of 10,000 ± 100 pores were created. The subpopulation of transient large pores may explain the rapid transport of macromolecules into and out of cells during a pulse [39].

Bensalem et al. (2020) [36] revealed a clear relationship between the size of FITC-dextran molecules that penetrated the cell membrane of *Chlamydomonas reinhardtii* and the efficacy of PEF treatments. Specifically, smaller FITC-dextran molecules (3 kDa) could penetrate the cell membrane under reversible PEF conditions. In comparison, larger molecules (10, 40, and 70 kDa) required irreversible PEF or a combination of PEF and mechanical compression. This suggests that the size of molecules that can enter cells following PEF exposure is directly determined by the pore size created, which in turn depends on the PEF parameters (pulse intensity, duration, and number). Notably, the minimal pore radius estimated for reversible electroporation was 0.77 nm, while irreversible electroporation with longer pulse durations (10 µs) allowed the penetration of molecules with a radius of up to 5.11 nm [36].

### 3.4. The Effect of PEF on Bacterial Cell Size as a Function of Time After PEF Exposure

*E. coli* bacterial cells were suspended in 0.56 mM PBS to an optical density of 0.05 OD (600 nm) as described in Section 2.3. A portion of a bacterial suspension was inserted into the electroporator cell and exposed to PEF, followed by staining with PI. The cell size was examined at specified times after PEF exposure. Each analyzed sample contained approximately 50,000 cells, ensuring that the areas under the curves were equal.

**Figure 4:**
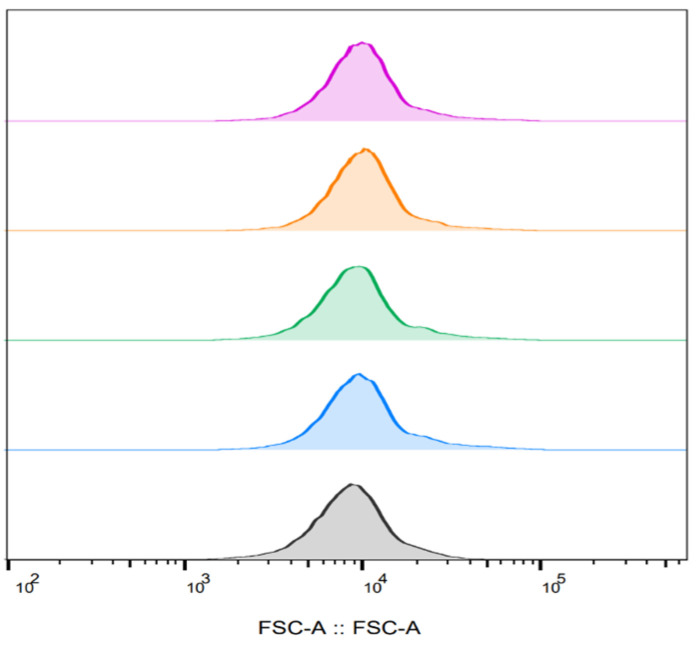
*E. coli* cell size as a function of the time after exposure to PEF. Bacterial suspension (0.5 OD 600 nm) that served as a control, which was not exposed to PEF (pink); bacterial suspension immediately after exposure (orange); 30 min after exposure (green); 60 min after exposure (blue); 90 min after exposure (gray).

Figure 4 shows the *E. coli* bacterial cell size immediately after PEF treatment (0 min) and after 30, 60, and 90 min. No change in cell size was found as a function of time after exposure to PEF. In addition, no change in cell size was observed even when compared to the control (unexposed) cells. The size of the cell was 9 × 10^3^ FSC.

The FSC value indicates bacterial cell size, where higher FSC values correspond to larger cells [40]. However, the relationship between cell size and FSC does not always hold, as cell morphology and type can also affect FSC values [41]. Cell volume changes following exposure to PEF are a critical aspect of electroporation, influenced by membrane permeability, pore dynamics, and overall cell viability. These volume alterations are primarily governed by osmotic gradients established between the intracellular and extracellular environments, particularly when the membrane becomes transiently permeable to solutes. Nesin et al. (2011) examined cell volume changes resulting from water uptake or expulsion, which occur as a consequence of solute movement outside and inside the cell. For both the 60 and 600 ns exposures, cell volume increased with increasing ns electric pulse intensity [35]. Radius changes in yeast cells were seen after PEF treatments (6 and 10 kV/cm); pulsed electric field application increased the nontreated cell radius by 2.4 μm (from 5.0 ± 0.1 μm to 7.4 ± 0.16 μm) [28]. Vaessen et al. (2019) [41] assessed the cell size of *Lactobacillus plantarum* under electric fields of 7.5, 10, and 12.5 kV/cm. The data revealed that the electroporation treatment did not affect the FSC values of the reversible cell fraction. However, high FSC values were obtained for the irreversible fraction. The results suggest that the larger cells in the population are more strongly affected by the PEF treatment, compared to the smaller cells [41].

### 3.5. Bacterial Viability

**Figure 5:**
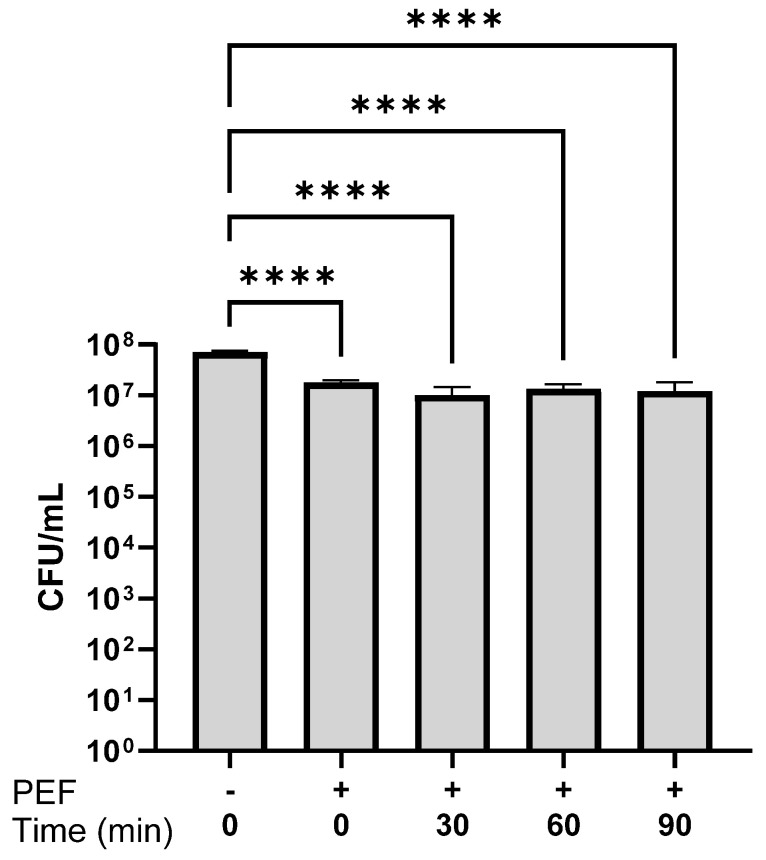
The CFU/mL in *E. coli* suspensions (0.1 OD 600 nm) exposed to PEF immediately after exposure 0. And 30, 60, and 90 min after PEF exposure, compared with the control suspension that was not exposed to PEF (“−0”). An ANOVA test was conducted to ascertain statistical significance. The data represent the mean ± SD of n = 4. **** *p* < 0.0001.

Exposing bacteria to PEF causes the creation of pores in the cell membrane, as observed in our experiments and previous studies [20,32,42]. To understand if the PEF conditions that were used in this study led to bacterial death, bacterial viability was examined by calculating the CFU. *E. coli* bacteria were suspended in 0.5 mM PBS to an optical density of 0.1 OD (600 nm). A portion of each bacterial suspension was inserted into the electroporator cell and exposed to PEF, as performed in all the experiments. Cell viability was checked immediately after exposure to PEF (time 0) and after incubation for 30, 60, and 90 min. After serial dilutions, the bacterial suspension was seeded on BH agar. The same procedure was performed for a PEF-nontreated bacterial suspension, which served as a control. The results are shown in Figure 5.

The CFU/mL of *E. coli* immediately after PEF exposure (0 min) was 1.78 × 10^7^ CFU/mL, whereas in the control suspension that was not exposed to PEF, it was 7.1 × 10^7^ CFU/mL. Exposure to PEF thus led to an immediate decrease of about half an order of magnitude. The bacterial counts of *E. coli* exposed to PEF after 30–90 min were 1.01 × 10^7^ CFU/mL and 1.2 × 10^7^ CFU/mL, respectively.

Emanuel et al. (2021) [13] examined the viability of PEF-treated *Staphylococcus aureus* and *P. putida* suspended in PBS (0–0.54 mM) at a final optical density of 0.01 at 600 nm. The bacteria were exposed to PEF (2.9 kV/cm, 100 Hz, square pulse shape) with a duration of 10 µs. The results showed that total eradication occurred only after 2 h when the bacteria were suspended in 0.54 mM PBS. At lower PBS concentrations, a bacterial reduction of between 0 and 2.5 log was observed [13]. Gędas et al. (2025) [43] analyzed the differentially expressed genes of *E. coli* (7.6 ± 0.2 log CFU/mL) inoculated in strawberry nectar after exposure to moderate-intensity PEF. Despite no microbial inactivation being revealed after PEF treatment, strong transcriptomic responses were observed, particularly in genes related to membrane integrity and metabolic activity [43].

## 4. Conclusions

Moderate PEF is a promising technology for the prevention of microorganisms in milk, milk product processing, poultry, eggs, juices, and other liquid foods. Recently available research papers have explored PEF technology not only to facilitate the inactivation of microorganisms but also to alleviate the pressing need for juice extraction from plants, intensify the drying and dehydration processes of food, and extract antioxidant compounds such as protein, polyphenols, chlorophyll, and carotenoids [44,45,46,47,48].

In our study, we examined environmental parameters that may affect *E. coli* membrane permeability, enabling the use of moderate PEF (3.3 kV cm^−1^) to extract substances from bacterial cells. The *E. coli* membrane permeability was examined as a function of time after PEF exposure. PI permeability percentages at a given time following PEF exposure in the different densities of bacterial suspensions revealed that as the bacterial density increased, there was a decrease in PI permeability. Membrane permeability was also examined in different PBS concentrations. The results showed that at a relatively low optical density (0.05), a linear correlation existed between PBS concentrations and PI permeability. At the higher bacterial densities of 0.1. and 0.5 OD, this phenomenon was not as clear. Examination of the bacterial membrane permeability with different molecular weights of fluorescein isothiocyanate (FITC)-dextran (4, 70, and 250 kDa) revealed that the highest percentage of 4 kDa FITC-dextran permeability was obtained 90 min after exposure, while the permeability of 70 kDa FITC-dextran at that time was about 2-fold less in comparison. The permeability of the 250 kDa FITC-dextran was not significant compared to the PEF-nontreated cells. The PEF parameters used in this study did not influence the bacterial cell size and viability. We assume that the results of this study may contribute to the implementation of PEF use in the food industry. However, more experiments should be conducted on other cell types, such as Gram-positive bacteria and yeast, since differences in cell envelope or size may influence cell susceptibility to PEF exposure.

## Figures and Tables

**Table 1:** The conductivity of the PBS solutions and the bacterial suspensions in the PBS.

0.5	0.1	0.05	0	OD 600 nm
G (µS/cm)				PBS (mM)
285 ± 0.1	278 ± 0.03	457.33 ± 2.5	445.33 ± 0.57	0.125
534 ± 0.04	524 ± 0.02	576.67 ± 3.51	647.67 ± 0.57	0.25
1061.5 ± 0.85	1104.5 ± 0.19	911.33 ± 17.78	1168 ± 3.6	0.5
1881 ± 0.61	1887.5 ± 0.29	1944 ± 8.89	1956 ± 7	1
3430 ± 0.42	3750 ± 0.14	3943.33 ± 15.27	3956.67 ± 5.78	2

## Data Availability

The original contributions presented in the study are included in the article/Supplementary Materials, further inquiries can be directed to the corresponding author.

## Supplementary Materials

The following supporting information can be downloaded at: https://www.mdpi.com/article/10.3390/microorganisms13081925/s1, File S1: Schematic drawing; File S2: Photos of electroporator chamber and the instrument.
