# Supplementary material for: The Influence of Moderate Electroporation on E. coli Membrane Permeability
Source: Microorganisms. 2025 Aug 18;13(8):1925. doi: 10.3390/microorganisms13081925 (PMC12388641; doi:10.3390/microorganisms13081925)
Supplement: Supplementary file 1 [file microorganisms-13-01925-s001.zip › File S1 Schematic drawing.pdf]

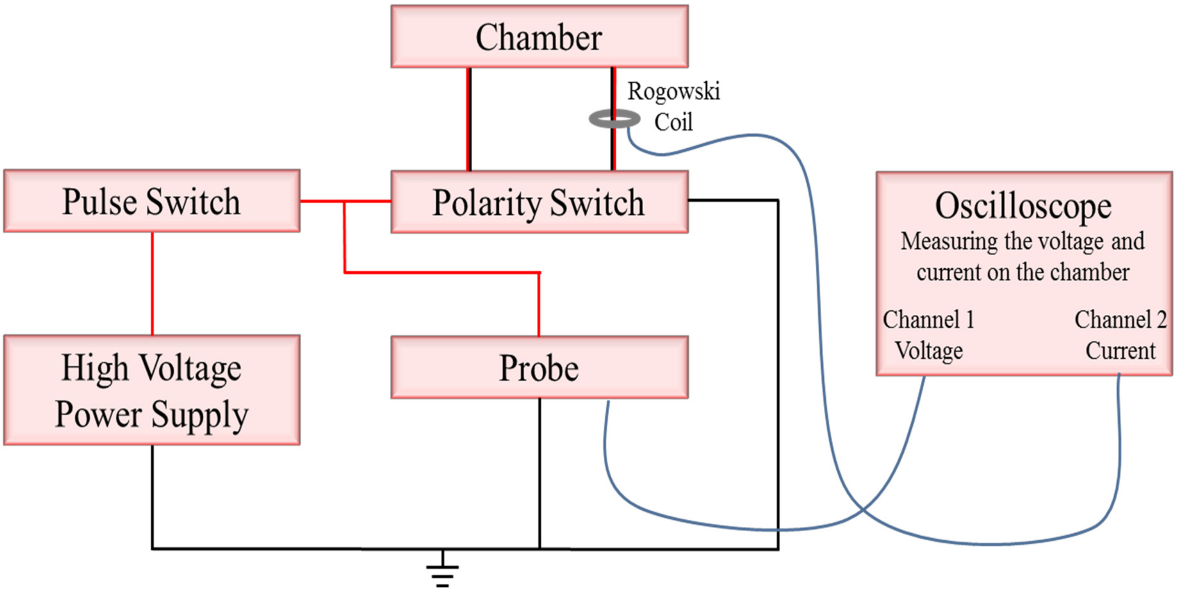

Figure S1. High voltage generator and the electronic circuit

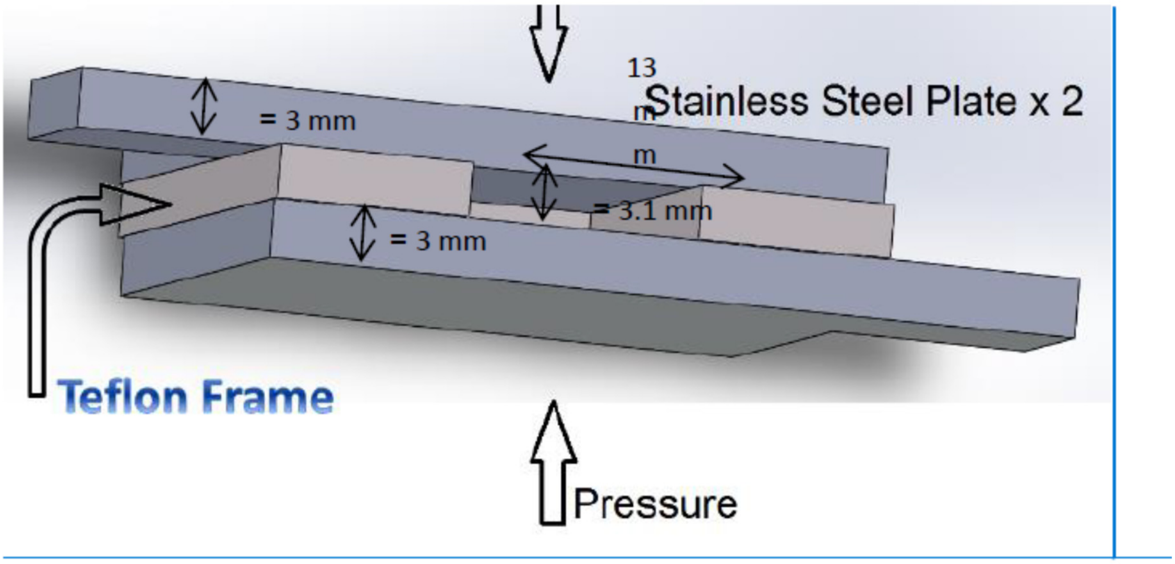

Figure S2. Schematic drawing of the electroporator chamber
