# Supplementary material for: The Influence of Moderate Electroporation on E. coli Membrane Permeability
Source: Microorganisms. 2025 Aug 18;13(8):1925. doi: 10.3390/microorganisms13081925 (PMC12388641; doi:10.3390/microorganisms13081925)
Supplement: Supplementary file 1 [file microorganisms-13-01925-s001.zip › File S2 Photos of electroporator chamber and the instrument.pdf]

Supplementary material S2

Photos of electroporator chamber and the instrument:

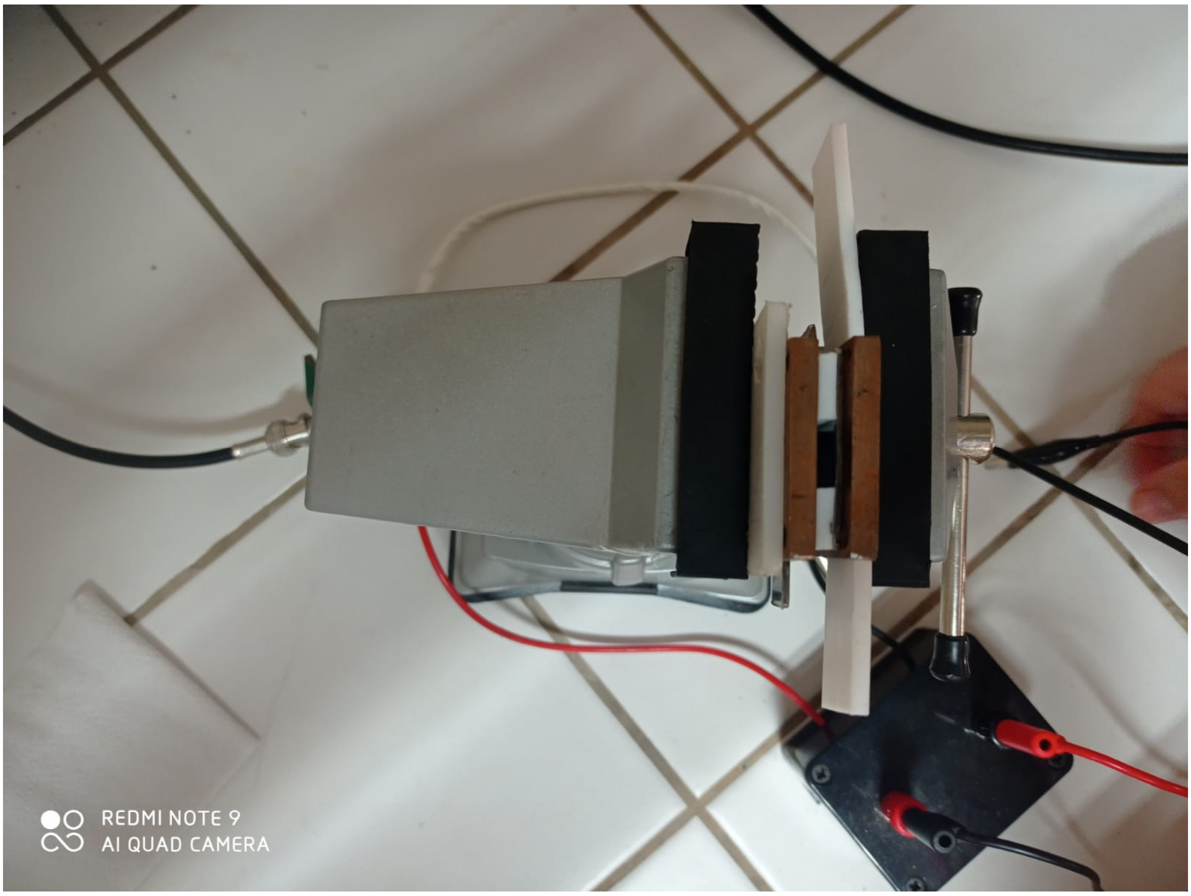

Figure S1. The electroporator chamber above view.

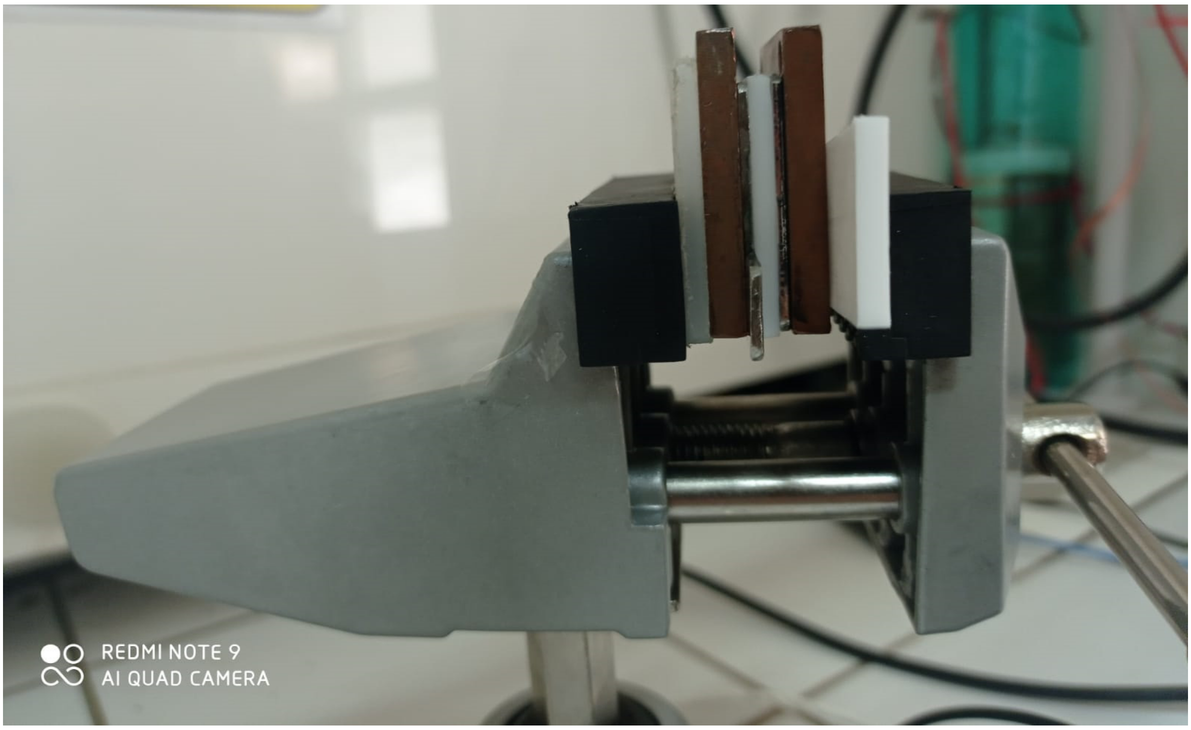

Figure S2. The electroorator chamber side view.

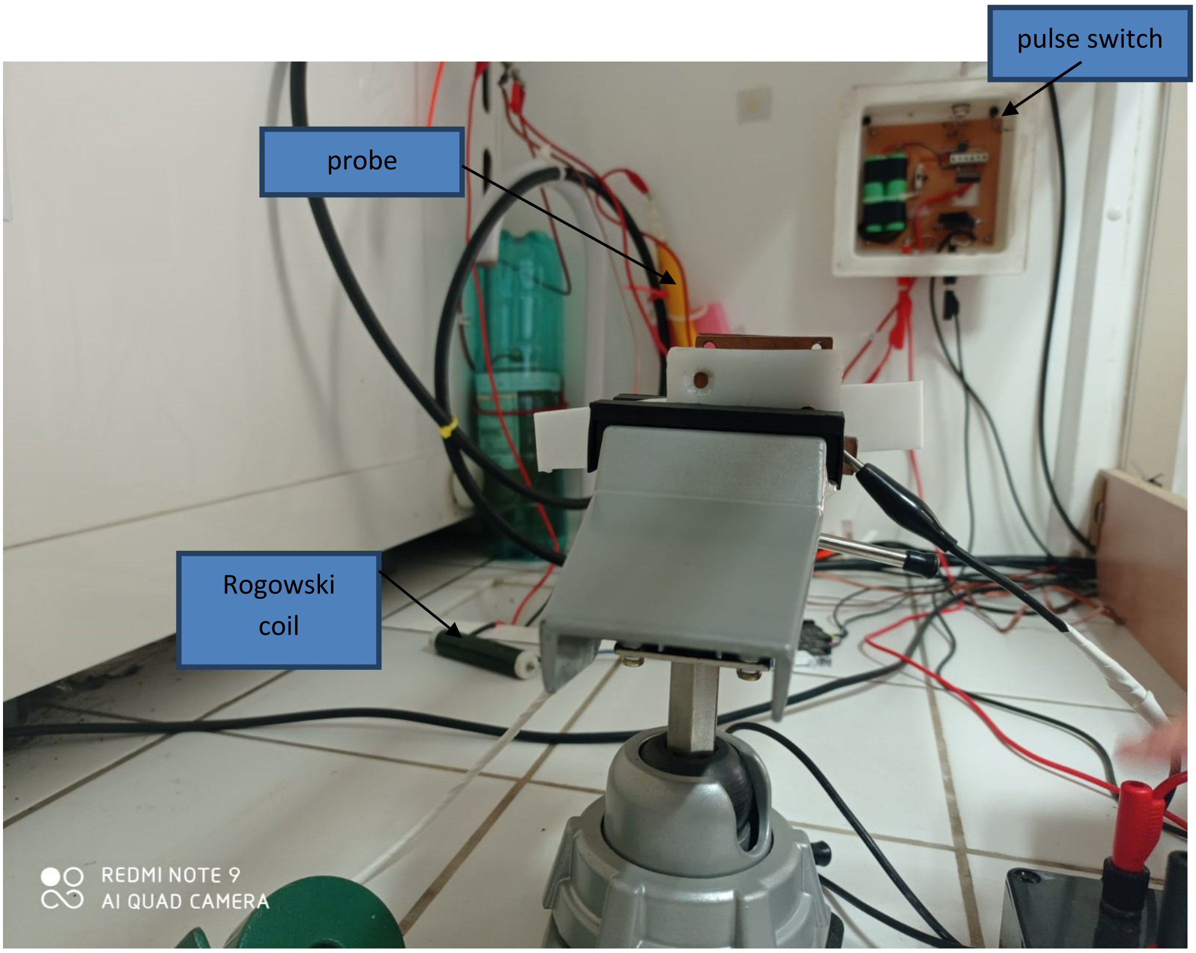

Figure S3. The instrument

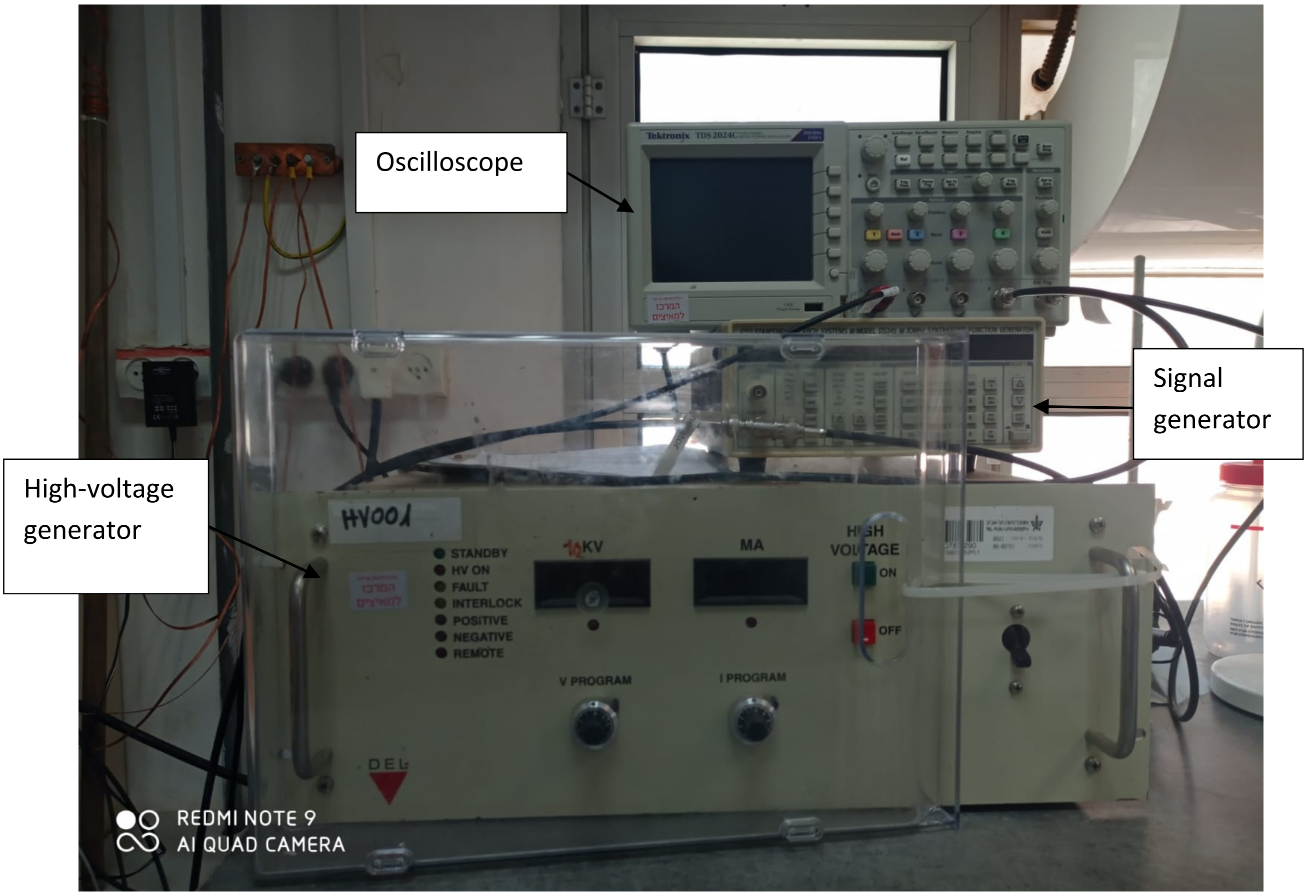

Figure S4. The instrument

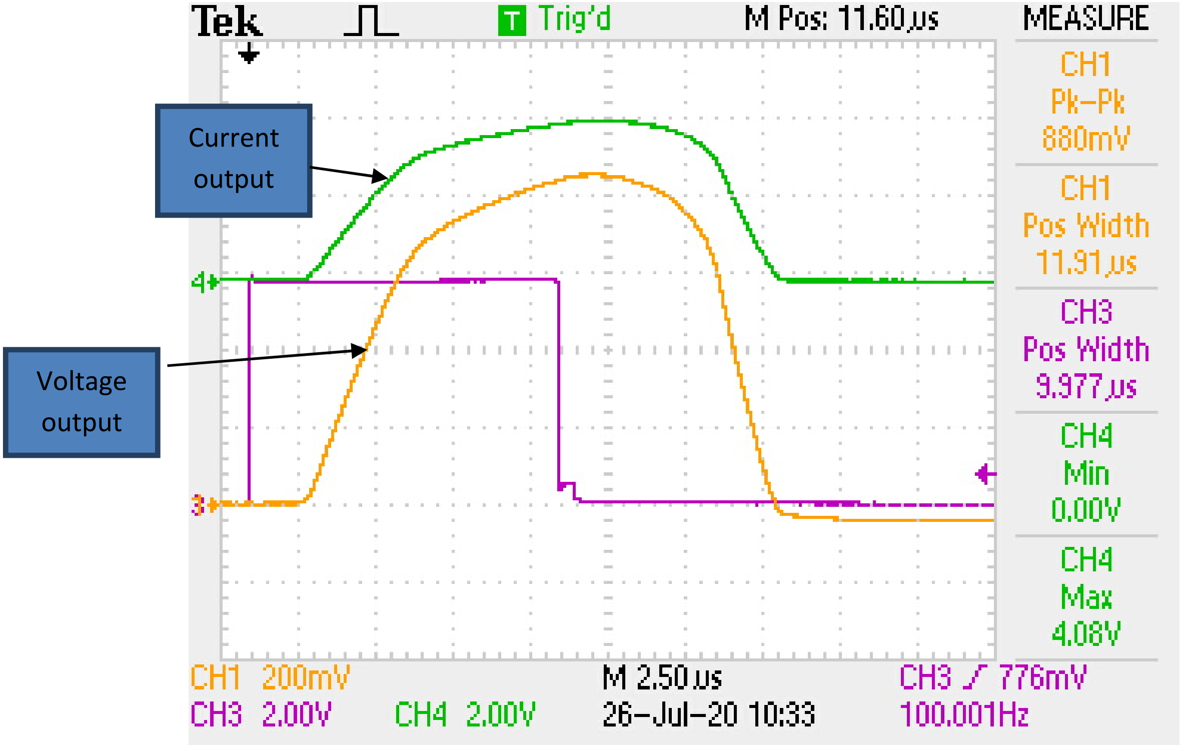

Figure S5. The shape (voltage and current) of the pulse on the cuvette filled with PBS (the solution with the highest conductivity).
